# Associations of built food environment with body mass index and waist circumference among youth with diabetes

**DOI:** 10.1186/1479-5868-9-81

**Published:** 2012-06-29

**Authors:** Archana P Lamichhane, Robin Puett, Dwayne E Porter, Matteo Bottai, Elizabeth J Mayer-Davis, Angela D Liese

**Affiliations:** 1 Department of Epidemiology and Biostatistics, Arnold School of Public Health, University of South Carolina, 921 Assembly Street, Columbia, SC, 29208, USA; 2 Department of Environmental Health Sciences, Arnold School of Public Health, University of South Carolina, Columbia, SC, USA; 3 South Carolina Cancer Prevention and Control Program, Arnold School of Public Health, University of South Carolina, Columbia, SC, USA; 4 Department of Nutrition, Gillings School of Global Public Health and School of Medicine, University of North Carolina at Chapel Hill, Chapel Hill, NC, USA

**Keywords:** Accessibility, Availability, Adiposity, BMI z-score, Waist circumference, Built food environment, Fast food outlet, Supermarket

## Abstract

**Background:**

Youth with diabetes are at increased risk for obesity and cardiovascular disease complications. However, less is known about the influence of built food environment on health outcomes in this population. The aim of this study was to explore the associations of accessibility and availability of supermarkets and fast food outlets with Body Mass Index (BMI) z-score and waist circumference among youth with diabetes.

**Methods:**

Information on residential location and adiposity measures (BMI z-score and waist circumference) for 845 youths with diabetes residing in South Carolina was obtained from the South Carolina site of the SEARCH for Diabetes in Youth study. Food outlets data obtained from the South Carolina Department of Health and Environmental Control and InfoUSA were merged based on names and addresses of the outlets. The comprehensive data on franchised supermarket and fast food outlets was then used to construct three accessibility and availability measures around each youth’s residence.

**Results:**

Increased number and density of chain supermarkets around residence location were associated with lower BMI z-score and waist circumference among youth with diabetes. For instance, for a female child of 10 years of age with height of 54.2 inches and weight of 70.4 pounds, lower supermarket density around residence location was associated with about 2.8–3.2 pounds higher weight, when compared to female child of same age, height and weight with highest supermarket density around residence location. Similarly, lower supermarket density around residence location was associated with a 3.5–3.7 centimeter higher waist circumference, when compared to residence location with the highest supermarket density. The associations of number and density of chain fast food outlets with adiposity measures, however, were not significant. No significant associations were observed between distance to the nearest supermarket and adiposity measures. However, contrary to our expectation, increased distance to the nearest fast food outlet was associated with higher BMI z-score, but not with waist circumference.

**Conclusions:**

Food environments conducive to healthy eating may significantly influence health behaviors and outcomes. Efforts to increase the availability of supermarkets providing options/selections for health-promoting foods may significantly improve the dietary intake and reduce adiposity among youth with diabetes.

## Background

The escalating prevalence of overweight among children and adolescents, which tripled between 1980 and 2002 [1,2], is a leading public health problem in the United States [3,4]. Compared to healthy youth, those with diabetes are at higher risk for development of cardiovascular risk factors. Contrary to general perceptions, youth with type 1 diabetes exhibit levels of overweight and obesity comparable to general youth populations [5]. Furthermore, type 2 diabetes has recently emerged in youth, with the vast majority of those affected having extremely high body mass index (BMI) values [5].

To date, most epidemiologic studies exploring the built food environment and health outcomes have focused on BMI, overweight or obesity among adults [6-10] with very few reports on children and adolescents [11-14]. Only one study has been conducted on the impact of the food environment on persons with diabetes [15]. Previous studies have reported poor dietary intake [16] and higher likelihood of cardiovascular risk factors including obesity [5,17] among youth with diabetes, despite an individual-level effort for diabetes management (such as medical nutrition therapy). Furthermore, the findings on lower availability of recommended foods such as whole grains, fruits, vegetables etc. for those with diabetes in low-income minority neighborhoods as an environmental barrier/predictor of meeting dietary recommendations for reducing diabetes complications and maintaining good health [18], suggest some causal link. However, the associations still need to be clarified given the dearth of data among population with diabetes.

One of the major concern of previous research assessing the influence of the built food environment is the characterization of the food environment, which has largely been limited to one environmental attribute at a time [6-8,14]. However, the availability of multiple different food outlet types in the same neighborhood [10], emphasizes the need for evaluation of the influence of these various outlet types. Separate evaluation of these food outlets can lead to false findings when the outlets are clustered in same geographic space. Thus, we aimed to explore the associations of accessibility and availability of supermarkets and fast food outlets with BMI z-score and waist circumference, in a high risk population of youth with diabetes (type 1 and type 2 diabetes) from South Carolina (SC). Distance to nearest food outlets from youth’s residence represented accessibility, and number and density of food outlets around youth’s residence represented availability and accessibility/availability measures, respectively. We evaluated the associations of supermarket and fast food outlet accessibility and availability with each adiposity measure separately and simultaneously.

## Methods

### Study design

Details on the SEARCH for Diabetes in Youth Study, hereafter called the SEARCH study, have been published [19]. SEARCH is a multi-center, multi-ethnic, population-based observational study of that ascertained prevalent non-gestational cases of physician-diagnosed diabetes in youth aged <20 years in 2001 and continues with the ascertainment of incident cases through the present. This study is limited to the South Carolina (SC) SEARCH site, which is one of the six clinical sites participating in SEARCH study. The SC SEARCH site includes youth with prevalent diabetes in 2001 (four counties) and newly diagnosed cases in 2002 and beyond (statewide). Data were collected during the initial patient survey and in-person clinic visits. Specifically, data on anthropometric measures was collected during baseline clinic visit for prevalent (2001) and incident (2006) cases, and baseline and follow-up 1 visit (12 months) for incident (2002–2005) cases by trained and certified SEARCH staff.

Youth with diabetes who participated in the SC site of the SEARCH study between 2001 and 2006 and who had at least one time point of anthropometric measures were eligible for this study. This study was reviewed and was approved by University of South Carolina’s Institutional Review Board.

### Individual-level characteristics

Height, weight and waist circumference were measured twice according to standardized protocols. Height was measured using a wall-mounted stadiometer or, if a home visit, a portable stadiometer. Weight was measured using an electronic, portable scale. Waist circumference was measured just above the uppermost lateral border of the right iliac crest following National Health and Nutrition Examination Survey protocol.

We obtained anthropometric measures at the baseline and two follow-up clinic visits. Age- and sex- specific BMI z-score were then calculated using the 2000 Centers for Disease Control and Prevention (CDC) growth chart [20] with interpolations made for youth >20 years at the time of the measurement.

Age at in-person clinic visit, race/ethnicity, gender and parental education were considered. Race/ethnicity was categorized as Non-Hispanic White (NHW), African American (AA)/others. Physician-diagnosed diabetes was categorized into type 1 and type 2 diabetes.

### Neighborhood-level characteristics

Census tract-level population for SC obtained from the United States Census Bureau 2000 Summary File 1 (SF1) [21] was used to calculate Census tract specific population density and was assigned to each youth based on his/her tract of residence.

### Built food environment data

We selected two different outlet types for our study based on food purchase options/selections they provide [9,10]. We defined supermarkets as large corporate-owned franchised food stores selling groceries, including fresh produce and meat, which differ from grocery stores and smaller non-corporate owned food stores [6] and included Bi-lo, Publix, WalMart, IGAs etc. Previous research has shown that chain supermarkets provide a large variety of healthful food at lower cost compared to other food stores [22]. Fast food outlets were defined as nationally or internationally known franchised limited service restaurants that sell inexpensive, quickly served foods such as hamburgers, and fried chicken [7] with payment made prior to receiving food, and had limited or no wait staff [23]. These outlets included Bojangles’, McDonald’s etc.

Data on food outlets including their geocoordinates were obtained from SC Department of Health and Environmental Control, SCDHEC (obtained in August 2008) and InfoUSA Inc. (obtained in February 2009). Our decision of generating a comprehensive food outlet dataset using two data sources was based on our recent validation work in SC, which showed better sensitivity and positive predicted values (PPV) for both supermarkets/grocery stores (sensitivity-86% and PPV-86%) and limited service restaurants (including fast food outlets) (sensitivity-93% and PPV-93%) [24]. After substantial data cleaning to remove spelling errors and duplicate entries, we identified a comprehensive list of 686 chain supermarkets and 2,624 chain fast food outlets in SC.

### Geocoding and built food environment measures

Addresses of youth with diabetes were geocoded to street address-level using Topographically Integrated Geographic Encoding and Referencing road files: TIGER 2000 and 2006 (obtained from the US Census Bureau) in ArcGIS 9.3 software (ESRI, Redlands,CA). The remaining addresses were geocoded using the “World Imagery” layer in ArcGIS. Out of a total 958 addresses, 899 had full street address information. A total of 845 (94%) with full street address could be successfully located and assigned geocoordinates.

Three measures of accessibility and availability [25] of supermarkets and fast food outlets for each participant were calculated using ArcGIS 9.3 and R 2.9.1 software. The total distance (in miles) to the nearest supermarket and fast food outlet, representing accessibility measure, was calculated using the shortest path along the road network from the residential location of each participant using network analyst extension in ArcGIS. Number of supermarkets, representing availability measure, was calculated in a 2-mile road network buffer from the residential location as a reasonable driving distance. Our study included urban to isolated rural areas, hence we also calculated supermarket availability using urbanicity-specific buffers (urban and large town areas: 2 miles, and sub-urban and small town/isolated rural areas: 6 miles) following Babey et al.[26] and used this information to conduct sensitivity analysis (i.e. to determine if the strength of associations of numbers of supermarkets with adiposity measures would change if we would have used considered different buffers to represent shopping environment in urban vs. rural environment). Fast food outlet availability was assessed only in close proximity (1-mile road network buffer) to the residential locations, with the assumption that youth are more likely to walk or bike to nearby outlets [27]. Density of supermarkets and fast food outlets (number per square mile), representing a combination of accessibility/availability measure, was estimated at youth’s residence by the Gaussian kernel density estimation method following 2 steps procedure: first a smoothed map to represent densities of outlets was generated. Bandwidths of 6-mile and 1-mile were selected as optimal bandwidth to generate the density surface for supermarkets and fast food outlets, respectively. The densities of food outlets for youth were then estimated by overlaying their residence locations on the kernel density map.

### Statistical analysis

We used generalized estimating equations (GEE) analyses to quantify the associations of the food environment measures with adiposity (BMI z-score and waist circumference), adjusting for the potential dependence of the measures taken repeatedly over time on each individual. Associations of the food environments and adiposity measures were assessed both separately and simultaneously. All analyses were adjusted for age at clinic visit, gender, race/ethnicity, diabetes type, diabetes duration and cohort year. Parental education and Census tract-level population density were included in sequential models. We started with stratified analyses to determine associations by diabetes type and found similar results. Hence, we proceeded with analysis which combined youth with type 1 and type 2 diabetes. All statistical analyses were performed in SAS 9.2 (SAS Institute Inc., Cary, NC, USA).

We tested the threshold effect and linearity assumption for all three major predictor variables (distance to nearest, number, and density of supermarkets and fast food outlets) included in our study. For each predictor variable, we tested for threshold effect by grouping the predictor into quartiles. We found no evidence of threshold effect for distance to nearest food outlets and number of food outlets in specific buffer based on the point estimates of quartile categories and the associated p-values. We observed possible threshold effect only for density of food outlets quartiles. We also tested for departure from linearity assumption for each predictor, by introducing second-order polynomial term (squared term) together with the linear term of each predictor. We found no evidence of departure from linearity for any of the predictors. Based on the results from threshold effect and linearity assumption analyses, we report GEE analyses results with simple continuous terms for predictors: distance to nearest food outlets and number of food outlets in specific buffer. Whereas, we report GEE results with quartile categories for density of food outlets.

## Results

### Participant profile

Table 1 presents the demographic and socioeconomic characteristics of our study sample. Compared to youth with type 2 diabetes, youth with type 1 diabetes were younger (Mean age = 10.8 vs. 15.6), had lower BMI (Mean BMI = 0.6 vs. 2.15) and had lower waist circumference (Mean waist circumference = 69.7 vs. 107.6). Furthermore, the majority of youth with type 1 diabetes was non-Hispanic white (69.8% vs. 21.0%), lived in households with incomes of $50,000 or more (43.2% vs. 8.5%), and had more than two-thirds parents with education beyond high school (71.9% vs. 46.0%).

**Table 1:** Baseline characteristics of youth with diabetes (N = 845; type 1 diabetes: 693 and type 2 diabetes: 152)

**Characteristics**	**Variables**	**All cases**		**Type 1 Diabetes**		**Type 2 Diabetes**	
		**Mean (SD) or %**	**Range**	**Mean (SD) or %**	**Range**	**Mean (SD) or %**	**Range**
Individual	Age at clinic visit	11.7 (4.7)	1.2,22.7	10.8 (4.6)	1.2,22.2	15.6 (2.9)	8.2,22.7
	Gender (Female) %	54.3	-	51.7	-	66.4	-
	Race/ethnicity %						
	Non-Hispanic white	61.1	-	69.8	-	21.0	-
	African American or other	38.9	-	30.2	-	79.0	-
	Highest parental education* %						
	Less than High school	6.3	-	4.8	-	13.2	-
	High school graduate	24.3	-	21.2	-	38.2	-
	Some College thru Associate Degree	35.0	-	36.6	-	27.6	-
	Bachelors degree or more	32.3	-	35.3	-	18.4	-
	Household income* %						
	<$25,000	24.6	-	20.6	-	42.8	-
	$25,000–49,999	21.5	-	21.6	-	21.0	-
	$50,000–74,999	16.2	-	18.5	-	5.9	-
	$75,000+	20.7	-	24.7	-	2.6	-
	BMI z-score	0.8 (1.1)	-3.3,3.4	0.6 (1.0)	-3.3,3.4	2.15 (0.5)	-0.07,3.1
	Waist circumference (cm)	76.3 (20.2)	43.0,159.3	69.7 (13.7)	43.0,121.5	107.6 (16.5)	61.2,159.3
Neighborhod	Median household income ($)	40,941 (13,597)	11,842, 91,459	42,249.8 (13,738.2)	11,842.0, 91,459.0	34,975.6(11,170.6)	15,232.0,72,955.0
	Population density (per sq. mile)	886.1 (1,135.2)	11.9, 12,042.9	868.5 (1,126.5)	11.9, 12042.9	966.4(1,174.9)	18.1,6,363.3

* % does not add up to 100 due to missing information on few participants.

All analyses exploring the associations of accessibility and availability of food outlets with adiposity measures adjusted for these demographic and socio-economic factors,

### Accessibility and availability of food outlets

The average distance to nearest supermarket from youth’s residence was 2.9 miles (average distance to nearest fast food outlet was 2.6 miles) (Table 2). On average, the number of supermarkets for youth in 2-mile buffer around residence was 1.1 (number of fast food outlets in 1-mile buffers was 1.2). The density of supermarkets was 1.1 per square mile (density of fast food outlets was 1.7 per square mile). Distribution of food outlets accessibility and availability measures remained similar when data was analyzed by diabetes types (Table 2).

**Table 2:** Local food accessibility/availability measures for youth with diabetes (N = 845; type 1 diabetes: 693 and type 2 diabetes: 152)

**Food outlets**	**Accessibility/availability measures**	**All cases**		**Type 1 diabetes**		**Type 2 diabetes**	
		**Mean (SD)**	**Range**	**Mean (SD)**	**Range**	**Mean (SD)**	**Range**
Supermarket	Distance to nearest (miles)	2.9 (2.7)	0.0, 17.4	2.9 (2.6)	0.0, 15.6	3.1 (3.1)	0.2, 17.4
	Number in 2-mile buffer	1.1 (1.5)	0.0, 9.0	1.1 (1.6)	0.0, 9.0	1.2 (1.4)	0.0, 6.0
	Density (number per sq. mile)	1.1 (0.7)	0.0, 2.6	1.1 (0.7)	0.0, 2.5	1.1 (0.8)	0.0, 2.6
Fast food	Distance to nearest (miles)	2.6 (2.5)	0.0, 15.9	2.5 (2.4)	0.0, 15.9	2.9 (3.1)	0.2, 15.7
	Number in 1-mile buffer	1.2 (2.7)	0.0, 17.0	1.2 (2.7)	0.0, 17.0	1.4 (2.7)	0.0, 14.0
	Density (number per sq. mile)	1.7 (2.0)	0.0, 9.8	1.7 (2.0)	0.0, 9.2	1.8 (2.1)	0.0, 9.8

### Associations of supermarket accessibility/availability with adiposity

No significant associations were observed between distance to the nearest supermarket and BMI z-score and waist circumference (Table 3, Model 1–4).

**Table 3:** Associations of supermarket accessibility/availability measures with BMI z-score and waist circumference in sequentially adjusted models

**Adiposity/ Supermarket accessibility and availability measures**	**Model 1**^**a**^		**Model 2**^**b**^		**Model 3**^**c**^		**Model 4**^**d**^	
	**Estimated difference**	**95% CI**	**Estimated difference**	**95% CI**	**Estimated difference**	**95% CI**	**Estimated difference**	**95% CI**
*BMI z-score*								
Distance to nearest (miles)	0.015	−0.010, 0.041	0.012	−0.014, 0.037	0.007	−0.021, 0.035	−0.031	−0.078, 0.017
Number in 2-mile buffer	−0.057**	−0.099, -0.016	−0.052*	−0.094, -0.011	−0.054*	−0.100, -0.008	−0.054*	−0.105, -0.004
Density (number per sq. mile)								
Lowest density (Quartile 1)	0.289**	0.096, 0.482	0.264**	0.069, 0.458	0.321**	0.089, 0.553	0.256	−0.012, 0.524
Quartile 2	0.248*	0.050, 0.446	0.233*	0.036, 0.430	0.281*	0.059, 0.502	0.232	−0.001, 0.464
Quartile 3	0.135	−0.041, 0.311	0.127	−0.048, 0.302	0.156	−0.020, 0.361	0.131	−0.060, 0.323
Highest density (Quartile 4)	-	-	-	-	-	-	-	-
*Waist circumference*								
Distance to nearest (miles)	0.126	−0.161, 0.413	0.102	−0.182, 0.386	−0.020	−0.323, 0.282	−0.312	−0.757, 0.133
Number in 2-mile buffer	−0.419	−0.876, 0.039	−0.400	−0.863, 0.077	−0.215	−0.708, 0.277	−0.235	−0.764, 0.294
Density (number per sq. mile)								
Lowest density (Quartile 1)	3.635**	1.416, 5.855	3.348**	1.064, 5.633	3.520**	0.992, 6.048	3.262*	0.521, 6.004
Quartile 2	3.603**	1.310, 5.897	3.612**	1.316, 5.909	3.753**	1.281, 6.226	3.442**	0.918, 5.966
Quartile 3	1.291	−0.628, 3.211	1.241	−0.681, 3.162	1.328	−0.652, 3.307	1.156	−0.861, 3.172
Highest density (Quartile 4)	-	-	-	-	-	-	-	-

^a^adjusted for age at clinic visit, race/ethnicity, gender, cohort year, diabetes type, diabetes duration.

^b^Model 1+ parental education.

^c^Model 2+ population density.

^d^Model 3+ fast food accessibility/availability.

p-value: * < 0.05, ** < 0.01.

Each additional supermarket within a 2-mile network buffer was associated with a significantly lower BMI z-score (estimated difference: -0.054, 95% CI: -0.100, -0.008; Table 3, Model 3) even after adjusting for individual-level covariates and population density. Similarly, each additional supermarket within 2-mile buffer was also associated with lower waist circumference; however, the association did not reach statistical significance. Further adjustment for fast food outlet availability (Table 3, Model 4), and median household income of individual’s tract (result not shown), did not attenuate the association. The strength or magnitude of associations of number of supermarkets in specific network buffer with BMI z-score and waist circumference remained similar for both 2-mile buffer measure and urbanicity-specific buffer measure (analysis with measure in urbanicity-specific buffer was performed just for sensitivity analysis; result not shown).

Compared to the quartile with the highest number of supermarket per square mile (supermarket density), the last two quartiles with a lower number of supermarket per square mile were associated with significantly higher BMI z-score (Quartile 1: estimated difference: 0.321, 95% CI: 0.089, 0.553; Quartile 2: estimated difference: 0.281, 95% CI: 0.059, 0.502; Table 3, Model 3) even after adjustment for individual-level covariates and population density. For instance, for a female child of 10 years of age with height of 54.2 inches and weight of 70.4 pounds, a lower supermarket density around residence location was associated with about 2.8–3.2 pounds higher weight, when compared to female child of same age, height and weight with the highest supermarket density around residence location. Similarly, for a female child of 15 years of age with height of 63.7 inches and weight of 114.9 pounds, a lower supermarket density around residence location was associated with about 5.1–5.9 pounds higher weight, when compared to female child of same age, height and weight with the highest supermarket density around residence location. The strength of associations between supermarket density quartiles and BMI z-score got slightly attenuated after adjustment for fast food outlet density (Table 3, Model 4), and median household income of each individual’s tract (result not shown); and the association was only marginally significant.

Similarly, compared to the quartile with the highest density of supermarkets, the last two quartiles with a lower density of supermarkets were also associated with significantly higher waist circumference (Quartile 1: estimated difference: 3.520, 95% CI: 0.992, 6.048; Quartile 2: estimated difference: 3.753, 95% CI: 1.281, 6.226; Table 3, Model 3) even after adjustment for individual-level covariates and population density. For instance, a lower supermarket density around residence location was associated with a 3.5–3.7 centimeter higher waist circumference, when compared to residence location with the highest supermarket density. Further adjustment for fast food outlet availability (Table 3, Model 4), and median household income of each individual’s tract (result not shown) did not attenuate the association between supermarket density quartiles and waist circumference.

### Associations of fast food outlet accessibility/availability with adiposity

Contrary to our expectation, a significantly higher BMI z-score was observed for each mile (estimated difference: 0.027, 95% CI: 0.002, 0.053; Table 4, Model 3) increase in distance between fast food outlet and youth’s residence after adjusting for individual-level covariates and population density. The associations remained significant even after adjustment for supermarket proximity. No significant association was observed for distance to the nearest fast food outlet and waist circumference.

**Table 4:** Associations of fast food outlet accessibility/availability measures with BMI z-score and waist circumference in sequentially adjusted models

**Adiposity/ Fast food accessibility and availability measures**	**Model 1**^**a**^		**Model 2**^**b**^		**Model 3**^**c**^		**Model 4**^**d**^	
	**Estimated difference**	**95% CI**	**Estimated difference**	**95% CI**	**Estimated difference**	**95% CI**	**Estimated difference**	**95% CI**
*BMI z-score*								
Distance to nearest (miles)	0.032**	0.008, 0.056	0.029*	0.005, 0.052	0.027*	0.002, 0.053	0.052*	0.007, 0.098
Number in 1-mile buffer	−0.015	−0.041, 0.011	−0.013	−0.039, 0.013	−0.009	−0.036, 0.017	0.002	−0.027, 0.031
Density (number per sq. mile)								
Lowest density (Quartile 1)	0.233*	0.048, 0.419	0.209*	0.024, 0.394	0.246*	0.027, 0.464	0.136	−0.115, 0.388
Quartile 2	0.188	−0.002, 0.378	0.169	−0.021, 0.359	0.200	−0.013, 0.414	0.122	−0.101, 0.346
Quartile 3	0.056	−0.123, 0.235	0.042	−0.137, 0.221	0.060	−0.122, 0.243	0.044	−0.141, 0.229
Highest density (Quartile 4)	-	-	-	-	-	-	-	-
*Waist circumference*								
Distance to nearest (miles)	0.270	−0.030, 0.568	0.251	−0.044, 0.545	0.150	−0.162, 0.462	0.404	−0.054, 0.861
Number in 1-mile buffer	−0.128	−0.429, 0.173	−0.108	−0.410, 0.194	0.0001	−0.310, 0.310	0.047	−0.288, 0.383
Density (number per sq. mile)								
Lowest density (Quartile 1)	2.437*	0.137, 4.737	2.297	−0.026, 4.620	1.905	−0.737, 4.548	0.423	−2.304, 3.151
Quartile 2	2.375*	0.143, 4.616	2.270*	0.008, 4.533	1.935	−0.596, 4.467	0.900	−1.576, 3.376
Quartile 3	0.520	−1.534, 2.575	0.375	−1.695, 2.445	0.184	−1.935, 2.303	0.051	−2.070, 2.171
Highest density (Quartile 4)	-	-	-	-	-	-	-	-

^a^adjusted for age at clinic visit, race/ethnicity, gender, cohort year, diabetes type, diabetes duration.

^b^Model 1+ parental education.

^c^Model 2+ population density.

^d^Model 3+ supermarket accessibility/availability.

p-value: * < 0.05, ** < 0.01.

No significant associations were observed between the number of fast food outlets and BMI z-score and waist circumference of youth.

Compared to the quartile with the highest fast food outlet density, the quartile with the lowest fast food outlet density was associated with significantly higher BMI z-score (estimated difference: 0.246, 95% CI: 0.027, 0.464; Table 4, Model 3) after adjustment for individual-level covariates and population density. The association, however, became non-significant once adjusted for the supermarket availability. No significant associations were observed between fast food outlet density quartiles and waist circumference.

## Discussion

Findings from our study suggest that increased accessibility/availability of supermarkets may be associated with decreased BMI z-score and waist circumference among youth with diabetes. However, the question of whether fast food outlets, environment considered to serve energy-dense foods, influence adiposity remains inconclusive.

The inverse associations between number and density of supermarkets around residence locations, and adiposity measures were in the expected direction and in agreement with some previous studies [6,9,10,12]. This may be due to the availability of various options/selections of health-promoting foods including fruits/vegetables and low-calorie products in a competitive environment with larger numbers of chain supermarkets, which can promote healthy dietary intake and ultimately health outcomes. However, factors such as individual’s food shopping skills/practices [28], and individual’s purchasing behaviors and social perceptions [29] can equally influence the relationships of food environment with health behaviors and outcomes.

The magnitude of threshold effects we observed with supermarket density quartiles and adiposity measures were very striking. Significantly higher weight and waist circumference were observed for residence locations with a lower supermarket densities compared to the highest supermarket density locations. For instance, for a female child of 10 years of age with height of 54.2 inches and weight of 70.4 pounds, lower supermarket density around residence location was associated with about 2.8–3.2 pounds higher weight, when compared to female child of same age, height and weight with the highest supermarket density around residence location. Similarly, a lower supermarket density around residence location was associated with a 3.5–3.7 centimeter higher waist circumference, when compared to residence location with the highest supermarket density.

The direction and magnitude of associations of supermarket accessibility/availability with both adiposity measures: BMI z-score and waist circumference further support a promising relationship between built food environment and increasing obesity. Previous studies have reported waist circumference as one of the best indicator of abdominal obesity in adults[30] as well as in children and adolescents [31]. In addition, it is also reported as a better predictor of cardiovascular disease risk in children [32] compared to BMI. To our knowledge, only one study among adults has examined the relationship between fast food outlet availability and change in waist circumference [33]. The study found that a high density of fast food outlets was associated with significantly increased waist circumference but only among frequent fast food outlets users.

While our study provided a strong support for the inverse associations of number and density of supermarkets around residence locations with adiposity measures, the associations of number and density of fast food outlets with adiposity measures were not significant. Furthermore, contrary to our hypothesis, we found significantly higher BMI z-score the farther the youth resided from the nearest fast food outlet. This unexpected direction of the association can be attributed to the spatial co-occurrences/clustering of both food outlet types. Our study region showed that almost 78% of supermarkets had one or more fast food outlets within one-half mile of supermarket locations. Spatial co-occurrences of multiple outlets in geographic space has been suggested earlier [10]. Majority of individuals residing far from chain fast food outlets may also reside far from chain supermarkets, particularly in semi-rural and rural settings. In such instance, residents will have to depend upon the local small food outlets for frequent food purchases. Previous studies have reported fewer supermarkets and larger proportions of small stores and convenience stores in rural areas [34,35], which offer limited selection and lower quality products [36,37] including fast food items [38,39], when compared with their urban counterparts. Hence, increased access to these types of local food venues, which are not considered in our study, could have attributed to higher BMI z-score among our youth population even though they resided far from the chain fast food outlets.

Mixed results have been reported on the influence of fast food outlets on adiposity. Previous studies found that the increased availability and accessibility of fast food outlets significantly contributed to increase in BMI, overweight or obesity [9,14,33]. However, other studies found no associations [7,11,23]. Despite extensive evidence linking fast food consumption with high energy and fat intake, nutrient-poor food, and increased overweight and obesity[40]; these mixed results on the influence of fast food outlets, can be attributed to a number of factors . Researchers have used various geographic scales of analysis (from state to Census block group [9,12,33,41,42]) for food environment measures. Furthermore, studies varied in terms of the anchoring point where the fast food usage was measured. Studies among children and adolescents used home [11,23] and/or school [14,43] locations. Studies among adults mostly used home [7,9,33]; only one study used both home and work [7] locations. The one-location approach can be unrealistic, given the possibility of use of a fast food outlet at particular points in time and space when a person is in need of something to eat [7]. Home, therefore, as used in our study, may represent only one of the many locations of fast food usage, which could explain the lack of significant associations of fast food availability with adiposity. Particularly, among children and youth, consideration of both home and school fast food environments are important since both can equally influence the eating behavior and hence adiposity.

Our study has several limitations. First, the addresses are the contact addresses of the youth and may not represent the residential location. Similar to the majority of built food environment studies, the food environment data used in our study was collected several years after the individual-level data were collected. It is likely that some changes occurred in food environments during the study period, however, these changes would most likely be occurring independently of adiposity of our study population and thus lead to non-differential misclassification. One of the major concerns in previous epidemiologic studies exploring the impact of food environment has been the validity of the food outlets data from secondary data sources [44]. However, our recent validation work in SC showed better sensitivity and positive predicted values for both supermarkets/grocery stores and limited service restaurants, when the combination of SCDHEC and InfoUSA datasets were used [24]. Hence, consideration of both data sources in our study would have minimized the count error. Furthermore, for error in the food outlet database to bias our results, it would have to be correlated with individual’s adiposity, which is highly unlikely. Our study considered only franchised grocery stores and franchised fast food outlets and did not include other local non-chain outlets because of high chances of misclassification (e.g. convenience stores providing food high in sugar, salt and fat were classified as small local grocery stores by secondary data sources).

Our study contributes to the literature in several ways. First, we quantified the accessibility and availability of food outlets at individual-level, an approach that has been described as ‘cutting edge’ in built food environment study [45]. Second, we included youth from the entire state ranging from urban to isolated rural areas. Third, we explored the impact of food environment on adiposity among youth with diabetes by considering two different outlets types that provide different food purchase options/selections. Only a few studies have explored the associations of various types of food environments with health outcomes among adults [9,10] and among children and youth populations [11,43]. This two-fold approach can be an important aspect to consider in SC and other states which lack specific land-use zoning and are typified by clustering of retail locations around arterial roads with high traffic volume [46]. Fourth, our study population included wide age range and large proportion of African Americans. Finally, we included three accessibility and availability measures that allowed us to capture different dimensions of the food environment, including evaluation of immediate proximity, variety and diversity in order to explore their associations with adiposity.

## Conclusions

In summary, our study suggests that built food environment may be an important contextual factor that significantly influences health behaviors and outcomes among youth with diabetes above and beyond the individual-level risk factors. In particular, higher number and density of supermarkets around residence location may lower excess weight and waist circumference gain among children and youth with diabetes, even in presence of outlets providing easy fast food options. Hence, efforts to increase availability of and awareness about healthy foods can have potential health implications. A recent state indicator report on fruits and vegetables published by the Centers for Disease Control and Prevention also provides support for policy and environmental strategies to improve fruit and vegetable availability and consumption [47]. Particularly, efforts to promote farmers markets, and small grocery stores and convenience stores offer fruits/vegetables and other healthy options can be a better solutions in rural areas, which have fewer chain supermarkets [34,35].

The relationship of fast food outlet with BMI z-score and waist circumference, however, remains inconclusive. Contrary to our expectation, higher BMI z-score was observed farther an individual resided from the nearest chain fast food outlet. This association may have been a result of lower access to health-promoting foods and increased access to lower quality products including fast food items, particularly in rural settings. Hence, consideration of these small food venues in future studies may help capture the total food environment exposures and assess their influence on health behavior and outcomes.

The relationships of food environment and health behaviors and outcomes can also be affected by factors such as individual’s food shopping skills/practices [28] and individual’s purchasing behaviors, perceptions of the availability and prices of foods, and social perceptions [29]. However, this link has not been well documented. Hence, future studies should explore individual, social as well as environment factors to provide a more comprehensive understanding of the influences of food environment on health outcomes.

## Competing interests

The authors declare that they have no competing interests.

## Authors’ contributions

APL designed the study, collected data, performed data management and analysis, interpreted data and drafted the manuscript. RP contributed to the conception of the study, interpretation of data, and critically revised and edited the manuscript. DEP contributed to the conception of the study, and critically revised and edited the manuscript. MB contributed to the conception of the study, data analysis, interpretation of the data, and critically revised and edited the manuscript. EJM contributed to the conception of the study, interpretation of the data, and critically revised and edited the manuscript. ADL contributed to the design of the study, interpretation of the data and critically revised and edited the manuscript. All authors read and approved the final version of the manuscript.

## References

[B1] Ogden CL Flegal KM Carroll MD Johnson CL Prevalence and trends in overweight among us children and adolescents, 1999–2000 JAMA 2002 288 1728 1732 10.1001/jama.288.14.1728 12365956

[B2] Hedley AA Ogden CL Johnson CL Carroll MD Curtin LR Flegal KM Prevalence of overweight and obesity among US children, adolescents, and adults, 1999-2002 JAMA 2004 291 2847 2850 10.1001/jama.291.23.2847 15199035

[B3] Ebbeling CB Pawlak DB Ludwig DS Childhood obesity: public-health crisis, common sense cure The Lancet 2002 360 473 482 10.1016/S0140-6736(02)09678-2 12241736

[B4] Ogden CL Carroll MD Curtin LR Lamb MM Flegal KM Prevalence of high body mass index in US children and adolescents, 2007-2008 JAMA 2010 303 242 249 10.1001/jama.2009.2012 20071470

[B5] Liu LL Lawrence JM Davis C Liese AD Pettitt DJ Pihoker C Dabelea D Hamman R Waitzfelder B Kahn HS Prevalence of overweight and obesity in youth with diabetes in USA: the SEARCH for Diabetes in Youth Study Pediatr Diabetes 2010 11 4 11 10.1111/j.1399-5448.2009.00519.x 19473302

[B6] Morland K Diez-Roux A Wing S Supermarkets, other food stores, and obesity the atherosclerosis risk in communities study Am J Prev Med 2006 30 333 339 10.1016/j.amepre.2005.11.003 16530621

[B7] Jeffery RW Baxter J McGuire M Linde J Are fast food restaurants an environmental risk factor for obesity Int J Behav Nutr Phys Act 2006 3 2 10.1186/1479-5868-3-2 10.1186/1479-5868-3-2 16436207 PMC1397859

[B8] Mujahid MS Diez-Roux A Shen M Gowda D Sanchez B Shea S Jacobs DR Jackson SA Relation between neighborhood environments and obesity in the multi-ethnic study of atherosclerosis Am J Epidemiol 2008 167 1349 1357 10.1093/aje/kwn047 18367469

[B9] Morland KB Evenson KR Obesity prevalence and the local food environment Health Place 2009 15 491 495 10.1016/j.healthplace.2008.09.004 19022700 PMC4964264

[B10] Rundle A Neckerman KM Freeman L Lovasi GS Purciel M Quinn J Richards C Sircar N Weiss C Neighborhood food environment and walkability predict obesity in New York City Environ Health Perspec 2009 117 442 10.1289/ehp.11590 PMC2661915 19337520

[B11] Liu GC Wilson JS Qi R Ying J Green neighborhoods, food retail and childhood overweight: differences by population density Am J Health Promot 2007 21 317 325 10.4278/0890-1171-21.4s.317 17465177

[B12] Powell LM Auld MC Chaloupka FJ OÆMalley PM Johnston LD Associations between access to food stores and adolescent body mass index Am J Prev Med 2007 33 301 307 10.1016/j.amepre.2007.07.007 17884578

[B13] Seliske LM Pickett W Boyce WF Janssen I Density and type of food retailers surrounding Canadian schools: variations across socioeconomic status Health Place 2009 15 903 907 10.1016/j.healthplace.2008.11.001 19121973

[B14] Davis B Carpenter C Proximity of fast-food restaurants to schools and adolescent obesity Am J Public Health 2009 99 505 510 10.2105/AJPH.2008.137638 19106421 PMC2661452

[B15] Millstein RA Yeh HC Brancati FL Batts-Turner M Gary TL Food availability, neighborhood socioeconomic status, and dietary patterns among blacks with type 2 diabetes mellitus Medscape J Med 2009 11 15 19295936 PMC2654697

[B16] Mayer-Davis EJ Nichols M Liese AD Bell RA Dabelea DM Johansen JM Pihoker C Rodriguez BL Thomas J Williams D Dietary intake among youth with diabetes: The SEARCH for diabetes in youth study J Am Diet Assoc 2006 106 689 697 10.1016/j.jada.2006.02.002 16647326

[B17] Rodriguez BL Fujimoto WY Mayer-Davis EJ Imperatore G Williams DE Bell RA Wadwa RP Palla SL Liu LL Kershnar A Prevalence of cardiovascular disease risk factors in U.S. children and adolescents with diabetes: the SEARCH for diabetes in youth study Diabetes Care 2006 29 1891 1896 10.2337/dc06-0310 16873798

[B18] Horowitz CR Colson KA Hebert PL Lancaster K Barriers to buying healthy foods for people with diabetes: evidence of environmental disparities Am J Pub Health 2004 94 1549 1554 10.2105/AJPH.94.9.1549 15333313 PMC1448492

[B19] SEARCH Study Group SEARCH for diabetes in youth: a multicenter study of the prevalence, incidence and classification of diabetes mellitus in youth Control Clin Trials 2004 25 458 471 15465616 10.1016/j.cct.2004.08.002

[B20] Kuczmarski RJ Ogden CL Guo SS Grummer-Strawn LM Flegal KM Mei Z Wei R Curtin LR Roche AF Johnson CL CDC growth charts for the United States: methods and development Vital Health Stat 2000 11 1 190 12043359

[B21] Bureau of the Census Census 2000 summary file 1 (SF1) 2001 http://www.census.gov/prod/cen2000/doc/sf1.pdf

[B22] Liese AD Weis KE Pluto D Smith E Lawson A Food store types, availability, and cost of foods in a rural environment J Am Diet Assoc 2007 107 1916 1923 10.1016/j.jada.2007.08.012 17964311

[B23] Burdette HL Whitaker RC Neighborhood playgrounds, fast food restaurants, and crime: relationships to overweight in low-income preschool children Prev Med 2004 38 57 63 10.1016/j.ypmed.2003.09.029 14672642

[B24] Liese AD Colabianchi N Lamichhane AP Barnes TL Hibbert JD Porter DE Nichols MD Lawson AB Validation of 3 food outlet databases: completeness and geospatial accuracy in rural and urban food environments Am J Epidemiol 2010 172 1324 1333 10.1093/aje/kwq292 20961970 PMC2991633

[B25] Guagliardo MF Spatial accessibility of primary care: concepts, methods and challenges Int J Health Geographics 2004 3 3 10.1186/1476-072X-3-3 PMC394340 14987337

[B26] Babey SH Diamant A Hastert TA Goldstein H Designed for disease: the link between local food environments and obesity and diabetes 2008 UC Los Angeles, UCLA Center for Health Policy Research http://www.healthpolicy.ucla.edu/pubs/files/Designed_for_Disease_050108.pdf

[B27] Timperio A Crawford D Telford A Salmon J Perceptions about the local neighborhood and walking and cycling among children Prev Med 2004 38 39 47 10.1016/j.ypmed.2003.09.026 14672640

[B28] Hersey J Anliker J Miller C Mullis RM Daugherty S Das S Bray CR Dennee P Sigman-Grant M Olivia AH Food shopping practices are associated with dietary quality in low-income households J Nutr Educ 2001 33 Suppl 1 S16 S26 12857541 10.1016/s1499-4046(06)60066-3

[B29] Giskes K van Lenthe FJ Brug J Mackenbach JP Turrell G Socioeconomic inequalities in food purchasing: the contribution of respondent-perceived and actual (objectively measured) price and availability of foods Prev Med 2007 45 41 48 10.1016/j.ypmed.2007.04.007 17532036

[B30] Pouliot MC Despres JP Lemieux S Moorjani S Bouchard C Tremblay A Nadeau A Lupien PJ Waist circumference and abdominal sagittal diameter: best simple anthropometric indexes of abdominal visceral adipose tissue accumulation and related cardiovascular risk in men and women Am J Cardiol 1994 73 460 468 10.1016/0002-9149(94)90676-9 8141087

[B31] Taylor RW Jones IE Williams SM Goulding A Evaluation of waist circumference, waist-to-hip ratio, and the conicity index as screening tools for high trunk fat mass, as measured by dual-energy X-ray absorptiometry, in children aged 3-19 y Am J Clin Nutr 2000 72 490 495 10919946 10.1093/ajcn/72.2.490

[B32] Savva SC Tornaritis M Savva ME Kourides Y Panagi A Silikiotou N Georgiou C Kafatos A Waist circumference and waist-to-height ratio are better predictors of cardiovascular disease risk factors in children than body mass index Int J Obes Relat Metab Disord 2000 24 1453 1458 10.1038/sj.ijo.0801401 11126342

[B33] Li F Harmer P Cardinal BJ Bosworth M Johnson-Shelton D Moore JM Acock A Vongjaturapat N Built environment and 1-year change in weight and waist circumference in middle-aged and older adults: Portland Neighborhood Environment and Health Study Am J Epidemiol 2009 169 401 408 19153214 10.1093/aje/kwn398 PMC2726645

[B34] Kaufman P Rural poor have less access to supermarkets, large grocery stores Rural Dev Perspect 1999 13 19 26

[B35] Sharkey JR Measuring potential access to food stores and food-service places in rural areas in the U.S Am J Prev Med 2009 36 S151 S155 10.1016/j.amepre.2009.01.004 19285206

[B36] Donkin AJ Dowler EA Stevenson SJ Turner SA Mapping access to food in a deprived area: the development of price and availability indices Public Health Nutr 2000 3 31 38 10786721 10.1017/s1368980000000057

[B37] Bustillos B Sharkey JR Anding J McIntosh A Availability of more healthful food alternatives in traditional, convenience, and nontraditional types of food stores in two rural Texas counties J Am Diet Assoc 2009 109 883 889 10.1016/j.jada.2009.02.011 19394475

[B38] Creel JS Sharkey JR McIntosh A Anding J Huber JC Availability of healthier options in traditional and nontraditional rural fast-food outlets BMC Public Health 2008 8 395 10.1186/1471-2458-8-395 19040722 PMC2614433

[B39] Sharkey JR Johnson CM Dean WR Horel SA Focusing on fast food restaurants alone underestimates the relationship between neighborhood deprivation and exposure to fast food in a large rural area Nutr J 2011 10 10 10.1186/1475-2891-10-10 21266055 PMC3036605

[B40] Duerksen SC Elder JP Arredondo EM Ayala GX Slymen DJ Campbell NR Baquero B Family restaurant choices are associated with child and adult overweight status in Mexican-American families J Am Diet Assoc 2007 107 849 853 10.1016/j.jada.2007.02.012 17467384

[B41] Maddock J The relationship between obesity and the prevalence of fast food restaurants: state-level analysis Am J Health Promot 2004 19 137 143 15559714 10.4278/0890-1171-19.2.137

[B42] Mehta NK Chang VW Weight status and restaurant availability a multilevel analysis Am J Prev Med 2008 34 127 133 10.1016/j.amepre.2007.09.031 18201642 PMC2440344

[B43] Seliske LM Pickett W Boyce WF Janssen I Association between the food retail environment surrounding schools and overweight in Canadian youth Public Health Nutr 2009 12 1384 1391 10.1017/S1368980008004084 19087383

[B44] Oakes JM Masse LC Messer LC Work group III: methodologic issues in research on the food and physical activity environments: addressing data complexity Am J Prev Med 2009 36 S177 S181 10.1016/j.amepre.2009.01.015 19285211

[B45] White M Food access and obesity Obesity reviews 2007 8 99 17316311 10.1111/j.1467-789X.2007.00327.x

[B46] Hurvitz PM Moudon AV Rehm CD Streichert LC Drewnowski A Arterial roads and area socioeconomic status are predictors of fast food restaurant density in King County, WA Int J Behav Nutr Phys Act 2009 6 46 10.1186/1479-5868-6-46 19630979 PMC2724491

[B47] Centers for Disease Control and Prevention State indicator report on fruits and vegetables 2009 http://www.fruitsandveggiesmatter.gov/downloads/StateIndicatorReport2009.pdf

